# A Real-Time Analysis Method for Pulse Rate Variability Based on Improved Basic Scale Entropy

**DOI:** 10.1155/2017/7406896

**Published:** 2017-05-09

**Authors:** Yongxin Chou, Ruilei Zhang, Yufeng Feng, Mingli Lu, Zhenli Lu, Benlian Xu

**Affiliations:** ^1^School of Electrical and Automatic Engineering, Changshu Institute of Technology, Changshu 215500, China; ^2^Changshu No. 1 People's Hospital, Changshu, China; ^3^State Key Laboratory of Robotics, Shenyang Institute of Automation, Chinese Academy of Sciences, Shenyang 110014, China

## Abstract

Base scale entropy analysis (BSEA) is a nonlinear method to analyze heart rate variability (HRV) signal. However, the time consumption of BSEA is too long, and it is unknown whether the BSEA is suitable for analyzing pulse rate variability (PRV) signal. Therefore, we proposed a method named sliding window iterative base scale entropy analysis (SWIBSEA) by combining BSEA and sliding window iterative theory. The blood pressure signals of healthy young and old subjects are chosen from the authoritative international database MIT/PhysioNet/Fantasia to generate PRV signals as the experimental data. Then, the BSEA and the SWIBSEA are used to analyze the experimental data; the results show that the SWIBSEA reduces the time consumption and the buffer cache space while it gets the same entropy as BSEA. Meanwhile, the changes of base scale entropy (BSE) for healthy young and old subjects are the same as that of HRV signal. Therefore, the SWIBSEA can be used for deriving some information from long-term and short-term PRV signals in real time, which has the potential for dynamic PRV signal analysis in some portable and wearable medical devices.

## 1. Introduction

Electrocardiogram (ECG) signal has been used for many diseases to assist in diagnosis in a clinic. The subtle changes of heart beat periods are called heart rate variability (HRV). The continuous heart rate or continuous RR wave intervals extracted from ECG signal are denoted as heart rate variability (HRV) signal [1]. An increasing number of studies have shown that HRV is a useful quantitative indicator for assessing the balance between the cardiac sympathetic nervous system and the parasympathetic nervous system and can be engaged in the diagnosis and prevention of some cardiovascular diseases such as sudden cardiac death and arrhythmia [2–4]. Pulse signal or continuous blood pressure signal generated by the systolic and diastolic of heart contains abundant physiological and pathological information of the cardiovascular system [5, 6]. The subtle change of vessel pulse periods is denoted as pulse rate variability (PRV). The continuous pulse rate or continuous PP wave intervals extracted from pulse signal or continuous blood pressure signal are defined as PRV signal [7]. Because a heartbeat produces a vessel pulse, many studies show that PRV is a substitute for HRV to present the physiological and pathological changes of the cardiovascular system when the subjects are sleeping or testing, as well as in some nonstationary states [8–10]. In addition, due to the wide distribution of human vessels, the acquisition of a pulse signal is easier than that of an ECG signal. Therefore, the pulse signal is employed in many wearable and portable medical devices such as smart watches, wristbands, and smart glasses but not ECG signal [11, 12], and PRV signal has more practical values than HRV signal.

Because PRV signal has similar characteristics with HRV signal, the analysis methods of HRV signal are often employed to analyze PRV signal. These methods are divided into time domain methods, frequency domain methods, time-frequency domain methods, and nonlinear methods [13]. HRV signal and PRV signal generated by heartbeat are neither stochastic nor periodic; they are the results of many independent factors and have nonlinear properties. Thus, the nonlinear methods are more useful for analyzing HRV signal and PRV signal, and there are many nonlinear methods such as recurrence quantification analysis, detrended fluctuation analysis, the Lyapunov exponent, and information entropy analysis [14, 15]. Among them, the information entropy analysis is an effective tool to present the complexity of the nonlinear signal. The sample entropy (SampEn), the approximate entropy (ApEn), the sign series entropy analysis (SSEA), the base scale entropy analysis (BSEA), and so on are been used for analyzing HRV signal [13, 16–18]. However, because of the long time consumption of these methods, they are not suitable for the PRV signal in real time. The BSEA, proposed by Li and Ning, can effectively detect the complexity dissimilarity of short-term HRV signal (about 5 minutes) in different physiological or pathological states [17], while it is unknown whether the BSEA is suitable for analyzing pulse rate variability (PRV) signal, so far. In addition, the 5 minutes of HRV signal analysis is too long for some acute cardiovascular disease (ACVD), and its time consumption still needs to be improved.

Therefore, this study proposed an improved basic scale entropy on the basis of BSEA with the theory of sliding window iterative; we denote it as sliding window iterative basic scale entropy analysis (SWIBSEA). The BSEA and SWIBSEA are engaged in analyzing the measured PRV signals, and by the results of the experiments, the accuracy and time consumption are compared between BSEA and SWIBSEA. In addition, the structure of this paper is as follows: in Section 2, the theories of BSEA and SWIBSEA are presented and then the experimental data are introduced. The results are shown in Section 3. Then, the results are discussed in Section 4. The conclusion is given in the last section.

## 2. Methods and Materials

### 2.1. Basic Scale Entropy Analysis

The process of BSEA is as follows [16]: (1) a series of vectors are constructed from PRV signal, and for each vector, we compute their basic scale (BS). (2) The vectors are symbolized and classified according to BS, each of these categories is a heart or pulse beat mode. (3) Computing the probability of each beat mode, and getting entropy of their probabilities, the entropy is denoted as BSE.

For a PRV signal with the length of *N*, PP : {PP(*i*) : 1 ≤ *i* ≤ *N*,  *i* ∈ *N*^∗^}, the *m* consecutive data points are used to construct a vector:

(1) $$\begin{array}{c} X\left(i\right)=\left[PP\left(i\right),PP\left(i+1\right),\ldots ,PP\left(i+m-1\right)\right]. \end{array}$$

Thus, we will get *N* − *m* + 1 vectors which are denoted as temporal sequence vectors (TSVs). *m* is the length of TSV; the larger the value of *m*, the more complex of the beat mode that TSV expresses. For each TSV, the BS is defined by the root mean square (RMS) of the difference for two adjacent data points:

(2) $$\begin{array}{c} BS\left(i\right)=\sqrt{\frac{\sum_{j=1}^{m-1}{\left(PP\left(i+j\right)-PP\left(i+j-1\right)\right)}^{2}}{m-1}}, \end{array}$$

where BS(*i*) is the BS of the *i*th TSV.

Then, the BS is multiplied by a constant *α*; the result is as the standard for the vector symbolization. The (*N* − *m* + 1) × *m* TSVs {*X*(*i*)} are symbolized, and the results are named symbol sequence vectors (SSVs) and denoted as {*S*_*i*_(*j*)}, {*S*_*i*_(*j*) : 1 ≤ *i* ≤ *N* − *m* + 1, 0 ≤ *j* ≤ *m* − 1, *i* ∈ *N*^∗^, *j* ∈ *N*}. The symbolization process is as follows:

(3) $$\begin{array}{c} S_{i}\left(j\right)=\left\{\begin{array}{cc} 0, & \bar{\mu_{i}}<{PP}_{i}\left(j\right)\leq \bar{\mu_{i}}+\alpha \times BS\left(i\right)\\ 1, & {PP}_{i}\left(j\right)>\bar{\mu_{i}}+\alpha \times BS\left(i\right)\\ 2, & \bar{\mu_{i}}-\alpha \times BS\left(i\right)<{PP}_{i}\left(j\right)\leq \bar{\mu_{i}}\\ 3, & {PP}_{i}\left(j\right)\leq \bar{\mu_{i}}-\alpha \times BS\left(i\right) \end{array}\right., \end{array}$$

where $\bar{\mu_{i}}$ is the mean of the *i*th TSV. PP_*i*_(*j*) is the (*i* + *j*)th data points of {PP(*i*)} or is the (*j* + 1)th datum in the *i*th TSV. The symbols 0, 1, 2, and 3 are the labels of different scopes for PRV amplitude and are employed for probability calculation; their values are of no practical significance. *S*_*i*_(*j*) is the (*j* + 1)th datum in the *i*th SSV. *α* is used to control the value of BS and to adjust the division range of PRV amplitude, the way to choose the value of *α* is as [19].

After getting the SSVs, we compute the probability of each vector. There are 4 symbols, 0, 1, 2, and 3, to express the vector, so we can get 4*^m^* kinds of different SSVs, denoted by *π*. Each SSV is a heart or pulse beat mode. Then, we compute the probability of each beat mode in *N* − *m* + 1 SSVs:

(4) $$\begin{array}{c} p\left(\pi \right)=\frac{\#\left\{\left.t\right|PPG\left(t\right),\ldots ,PPG\left(t+m-1\right)\;has\;type\;\pi \right\}}{N-m+1}, \end{array}$$

where 1 ≤ *t* ≤ 4, *t* ∈ *N*^∗^, and # is the number of *π*. The beat state with probability 0 is denoted as “disabled mode.”

Therefore, we define BSE as

(5) $$\begin{array}{c} BSE\left(m\right)=-\sum p\left(\pi \right)\log_{2}p\left(\pi \right). \end{array}$$

The BSE can be used to describe the change of heartbeat mode. Obviously, 0 ≤ BSE(*m*) ≤ log_2_4^*m*^. When there is only one pulse mode, BSE(*m*) = 0. When there are 4*^m^* pulse modes, and each mode has equal probability, BSE(*m*) = log_2_4^*m*^ is the maximum. The larger the entropy value, the more complicated the heartbeat mode, whereas the smaller the entropy value, the simpler the heartbeat mode.

### 2.2. Sliding Window Iterative Basic Scale Entropy Analysis

**Figure 1:**
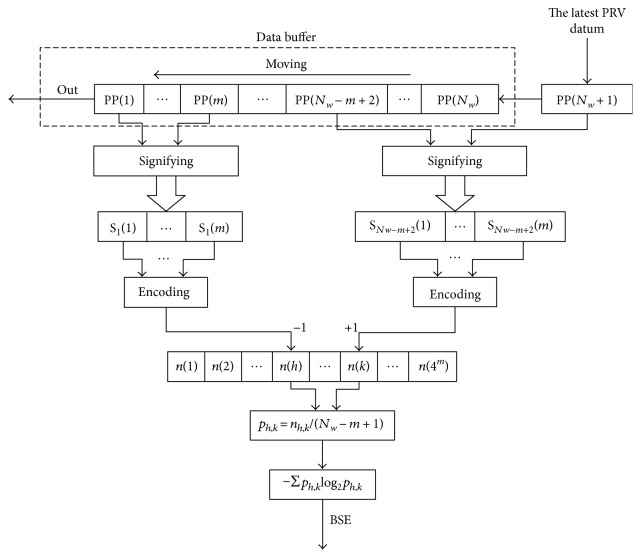
The process of SWIBSEA.

We improve the BSEA with the theory of sliding window iterative, and define the improved method as sliding window iterative base scale entropy analysis. The process is shown in Figure 1.

In Figure 1, a data buffer with the length of *N*_*w*_ is set to store PRV data points which are extracted from dynamic pulse signal. The process of SWIBSEA contains data updating and iterative. The PRV datum will be updated by the sliding window theory, and the BSE will be calculated with the iterative theory.

During data updating, we set 1 byte in buffer to store the latest PRV datum and denote it as PP(*N*_*w*_ + 1). Then, we delete the oldest PRV datum PP(1), and the data in higher addresses move to lower addresses, PP(*i*) = PP(*i* + 1). If we image the PRV signal as fixed, the buffer is like sliding forward in the data. This is the process of sliding window.

During iterative, the data in PP(1) and PP(*N*_*w*_) is only changed in data updating, so it is only needed to compute the entropy of SSVs corresponding to PP(1) and PP(*N*_*w*_), and then we will get the new BSE of all PRV data in buffer.

#### 2.2.1. Construct the Temporal Sequence Vectors

According to the theory of BSEA, we need to reconstruct the PRV data with the length of *N*_*w*_ to (*N*_*w*_ − *m* + 1) × *m* TSVs. In the process of updating data, only the data in PP(*N*_*w*_ + 1) and PP(1) are changed; thus, for speeding up the calculation, we only construct the TSVs corresponding to them.

(6) $$\begin{array}{c} X\left(1\right)=\left[PP\left(1\right),PP\left(2\right),\ldots ,PP\left(m\right)\right], \end{array}$$

(7) $$\begin{array}{c} X\left(N_{w}-m+2\right)=\left[PP\left(N_{w}-m+2\right),PP\left(N_{w}-m+3\right),\ldots ,PP\left(N_{w}+1\right)\right], \end{array}$$

where *X*(1) is the TSV of PP(1), *X*(*N*_*w*_ − *m* + 2) is the TSV of PP(*N*_*w*_ + 1).

#### 2.2.2. The Symbolization of Temporal Sequence Vectors *X*(*N*_*w*_ − *m* + 2) and *X*(1)

For *X*(1) computation, by (3),

(8) $$\begin{array}{c} S_{1}\left(j\right)=\left\{\begin{array}{cc} 0, & \mu_{1}<PP\left(j\right)\leq \mu_{1}+\alpha \times {BS}_{1}\\ 1, & PP\left(j\right)>\mu_{1}+\alpha \times {BS}_{1}\\ 2, & \mu_{1}-\alpha \times {BS}_{1}<PP\left(j\right)\leq \mu_{1}\\ 3, & PP\left(j\right)\leq \mu_{1}-\alpha \times {BS}_{1} \end{array}\right., \end{array}$$

where *μ*_1_ is the mean of *X*(1). BS_1_ is the base scale of *X*(1), by (2):

(9) $$\begin{array}{c} {BS}_{1}=\sqrt{\frac{\sum_{j=1}^{m-1}{\left(PP\left(j+1\right)-PP\left(j\right)\right)}^{2}}{m-1}}. \end{array}$$

Similarly, *X*(*N*_*w*_ − *m* + 2) is symbolized and denoted as {*S*_*N*_*w*_−*m*+2_(*j*)}, *j* = 1,…, *m* − 1.

#### 2.2.3. Encode the Symbol Sequence Vector

During the data updating, only the numbers of {*S*_1_(*j*)} and {*S*_*N*_*w*_−*m*+2_(*j*)} are changed; thus we only need to update their numbers. Moreover, for speeding up the calculation, we encode {*S*_1_(*j*)} and {*S*_*N*_*w*_−*m*+2_(*j*)} to generate their storage addresses. If we denote the addresses as *h* and *k* (as is shown in Figure 1), then

(10) $$\begin{array}{c} h=\overset{m}{\underset{j=1}{\sum }}S_{1}\left(j\right)\times 4^{m-j} \end{array}$$

and

(11) $$\begin{array}{c} k=\overset{m}{\underset{j=1}{\sum }}S_{N_{w}-m+2}\left(j\right)\times 4^{m-j}. \end{array}$$

#### 2.2.4. Entropy Calculation with Iterative Theory

We update the PRV data in buffer by sliding window theory; when getting a new PRV datum, the oldest PRV datum should be deleted. Thus, when computing entropy, we should just subtract the entropy of {*S*_1_(*j*)} and add the entropy of {*S*_*N*_*w*_−*m*+2_(*j*)} from the entropy before data updating. Meanwhile, the intermediate variables are also need update by iterative after each data updating.

As shown in Figure 1, we denote the numbers of {*S*_1_(*j*)} and {*S*_*N*_*w*_−*m*+2_(*j*)} as *n*′(*h*), *n*′(*k*) and *n*(*h*), *n*(*k*), the probabilities of {*S*_1_(*j*)} and {*S*_*N*_*w*_−*m*+2_(*j*)} as *p*′(*h*), *p*′(*k*) and *p*(*h*), *p*(*k*), and the entropies of {*S*_1_(*j*)} and {*S*_*N*_*w*_−*m*+2_(*j*)} as BSE′(*m*) and BSE(*m*) before and after updating PRV data, respectively. After data updating, *n*(*h*) should subtract 1 and *n*(*k*) should add 1. Then, *n*(*h*) = *n*′(*h*) − 1, *n*(*k*) = *n*′(*k*) + 1. The initial value of BSE′(*m*) = 0. In the iterative process of computing entropy, the antilogarithm of logarithm must be over 0; according to the changes of *n*′(*h*), *n*′(*k*), *n*(*h*), and  *n*(*k*), there are four kinds of iterative methods to computing BSE as follows:

(1) **n**(**h**) > 0, **n**(**k**) > 1, which means the beat modes that {*S*_1_(*j*)} and {*S*_*N*_*w*_−*m*+2_(*j*)} expressed all exist. Thus, during computing BSE, the antilogarithm of logarithm will be over 0. By (5),

(12) $$\begin{array}{c} BSE^{\prime }\left(m\right)=-\overset{M}{\underset{i=1}{\sum }}p^{\prime }\left(i\right)\log_{2\;}p^{\prime }\left(i\right)=-p^{\prime }\left(1\right)\log_{2}\;p^{\prime }\left(1\right)\cdots -p^{\prime }\left(h\right)\log_{2}\;p^{\prime }\left(h\right)\cdots -p^{\prime }\left(k\right)\log_{2}\;p^{\prime }\left(k\right)\cdots -p^{\prime }\left(M\right)\log_{2}\;p^{\prime }\left(M\right)=-p^{\prime }\left(h\right)\log_{2}\;p^{\prime }\left(h\right)\cdots -p^{\prime }\left(1\right)\log_{2}\;p^{\prime }\left(1\right)\cdots -p^{\prime }\left(M\right)\log_{2}\;p^{\prime }\left(M\right)\cdots -p^{\prime }\left(k\right)\log_{2}\;p^{\prime }\left(k\right), \end{array}$$

(13) $$\begin{array}{c} BSE\left(m\right)=-\overset{M}{\underset{i=1}{\sum }}p\left(i\right)\log_{2}\;p\left(i\right)=-p\left(1\right)\log_{2}\;p\left(1\right)\cdots -p\left(h\right)\log_{2}\;p\left(h\right)\cdots -p\left(k\right)\log_{2}\;p\left(k\right)\cdots -p\left(M\right)\log_{2}\;p\left(M\right)=-p\left(h\right)\log_{2}\;p\left(h\right)\cdots -p\left(1\right)\log_{2}\;p\left(1\right)\cdots -p\left(M\right)\log_{2}\;p\left(M\right)\cdots -p\left(k\right)\log_{2}\;p\left(k\right), \end{array}$$

where *M* = 4^*m*^.

Because only the modes of {*S*_1_(*j*)} and {*S*_*N*_*w*_−*m*+2_(*j*)} are changed, thus, −*p*(1)log_2_*p*(1) = −*p*′(1)log_2_*p*′(1),…, −*p*(*M*)log_2_*p*(*M*) = −*p*′(*M*)log_2_*p*′(*M*). With (12) and (13),

(14) $$\begin{array}{c} BSE\left(m\right)=BSE^{\prime }\left(m\right)-p\left(h\right)\log_{2}p\left(h\right)-p\left(k\right)\log_{2}p\left(k\right)+p^{\prime }\left(h\right)\log_{2}p^{\prime }\left(h\right)+p^{\prime }\left(k\right)\log_{2}p^{\prime }\left(k\right). \end{array}$$

Simplify (14):

(15) $$\begin{array}{c} BSE\left(m\right)=BSE^{\prime }\left(m\right)-\frac{n\left(h\right)}{N_{w}-m+1}\log_{2}\left(\frac{n\left(h\right)}{N_{w}-m+1}\right)-\frac{n\left(k\right)}{N_{w}-m+1}\log_{2}\left(\frac{n\left(k\right)}{N_{w}-m+1}\right)+\frac{n^{\prime }\left(h\right)}{N_{w}-m+1}\log_{2}\left(\frac{n^{\prime }\left(h\right)}{N_{w}-m+1}\right)+\frac{n^{\prime }\left(k\right)}{N_{w}-m+1}\log_{2}\left(\frac{n^{\prime }\left(k\right)}{N_{w}-m+1}\right)=BS^{\prime }\left(m\right)-\frac{n\left(h\right)}{N_{w}-m+1}\log_{2}\left(\frac{n\left(h\right)}{N_{w}-m+1}\right)-\frac{n\left(k\right)}{N_{w}-m+1}\log_{2}\left(\frac{n\left(k\right)}{N_{w}-m+1}\right)+\frac{n\left(h\right)+1}{N_{w}-m+1}\log_{2}\left(\frac{n\left(h\right)+1}{N_{w}-m+1}\right)+\frac{n\left(k\right)-1}{N_{w}-m+1}\log_{2}\left(\frac{n\left(k\right)-1}{N_{w}-m+1}\right)=BS^{\prime }\left(m\right)+\frac{n\left(h\right)}{N_{w}-m+1}\log_{2}\left(1+\frac{1}{n\left(h\right)}\right)+\frac{1}{N_{w}-m+1}\log_{2}\left(\frac{n\left(h\right)+1}{n\left(k\right)-1}\right)+\frac{n\left(k\right)}{N_{w}-m+1}\log_{2}\left(1-\frac{1}{n\left(k\right)}\right). \end{array}$$

Note that when the beat mode of {*S*_1_(*j*)} is the same as that of {*S*_*N*_*w*_−*m*+2_(*j*)} before and after data updating, the number of the beat mode that {*S*_1_(*j*)} expressed should subtract 1: *n*(*h*) = *n*′(*h*) − 1, and the number of the beat mode that {*S*_*N*_*w*_−*m*+2_(*j*)} expressed should add 1: *n*(*k*) = *n*′(*k*) + 1 = *n*(*h*) + 1 = *n*′(*h*). Then, (14) is

(16) $$\begin{array}{c} BSE\left(m\right)=BSE^{\prime }\left(m\right)+\frac{n\left(h\right)+1}{N_{w}-m+1}\log_{2}\left(\frac{n\left(h\right)+1}{N_{w}-m+1}\right)+\frac{n\left(h\right)}{N_{w}-m+1}\log_{2}\left(\frac{n\left(h\right)}{N_{w}-m+1}\right)-\frac{n\left(h\right)}{N_{w}-m+1}\log_{2}\left(\frac{n\left(h\right)}{N_{w}-m+1}\right)-\frac{n\left(h\right)+1}{N_{w}-m+1}\log_{2}\left(\frac{n\left(h\right)+1}{N_{w}-m+1}\right)=BSE^{\prime }\left(m\right). \end{array}$$

That means the entropy is not changed when data updating. Because the beat modes of {*S*_1_(*j*)} and {*S*_*N*_*w*_−*m*+2_(*j*)} are the same, the total number of beat modes remains unchanged.

(2) **n**(**h**) = 0, **n**(**k**) > 1, which means the beat mode {*S*_1_(*j*)} disappeared after data updating, and *n*′(*h*) = 1, *p*(*h*) = 0, *p*′(*h*) = 1/(*N*_*w*_ − *m* + 1). By (14),

(17) $$\begin{array}{c} BSE\left(m\right)=BSE^{\prime }\left(m\right)+\frac{1}{N_{w}-m+1}\log_{2}\left(\frac{1}{N_{w}-m+1}\right)+\frac{n\left(k\right)-1}{N_{w}-m+1}\log_{2}\left(\frac{n\left(k\right)-1}{N_{w}-m+1}\right)-\frac{n\left(k\right)}{N_{w}-m+1}\log_{2}\left(\frac{n\left(k\right)}{N_{w}-m+1}\right)=BSE^{\prime }\left(m\right)+\frac{n\left(k\right)}{N_{w}-m+1}\log_{2}\left(1-\frac{1}{n\left(k\right)}\right)-\frac{1}{N_{w}-m+1}\log_{2}\left(n\left(k\right)-1\right). \end{array}$$

(3) **n**(**h**) > 0, **n**(**k**) = 1, which means the beat mode of {*S*_*N*_*w*_−*m*+2_(*j*)} appeared the first time after data updating, and *n*′(*h*) = *n*(*h*) + 1, *n*(*k*) = 1, *n*′(*k*) = 0, *p*′(*k*) = 0, *p*(*k*) = 1/(*N*_*w*_ − *m* + 1). By (14),

(18) $$\begin{array}{c} BSE\left(m\right)=BSE^{\prime }\left(m\right)+\frac{n\left(h\right)+1}{N_{w}-m+1}\log_{2}\left(\frac{n\left(h\right)+1}{N_{w}-m+1}\right)-\frac{n\left(h\right)}{N_{w}-m+1}\log_{2}\left(\frac{n\left(h\right)}{N_{w}-m+1}\right)-\frac{1}{N_{w}-m+1}\log_{2}\left(\frac{1}{N_{w}-m+1}\right)=BSE^{\prime }\left(m\right)+\frac{n\left(h\right)}{N_{w}-m+1}\log_{2}\left(1+\frac{1}{n\left(h\right)}\right)+\frac{1}{N_{w}-m+1}\log_{2}\left(n\left(h\right)+1\right). \end{array}$$

(4) **n**(**h**) = 0, **n**(**k**) = 1, which means the beat mode of {*S*_1_(*j*)} disappeared, the beat mode of {*S*_*N*_*w*_−*m*+2_(*j*)} appeared the first time after updating data, and the total number of beat modes is unchanged. Then, *n*′(*h*) = 1, *n*′(*k*) = 0, *p*′(*k*) = *p*(*h*) = 0, *p*′(*h*) = *p*(*k*) = 1/(*N*_*w*_ − *m* + 1). By (14),

(19) $$\begin{array}{c} BSE\left(m\right)=BSE^{\prime }\left(m\right)+\frac{1}{N_{w}-m+1}\log_{2}\left(\frac{1}{N_{w}-m+1}\right)-\frac{1}{N_{w}-m+1}\log_{2}\left(\frac{1}{N_{w}-m+1}\right)=BSE^{\prime }\left(m\right). \end{array}$$

By (15), (16), (17), (18), and (19), we will obtain the BSE of PRV signal based on sliding window and iterative theory.

### 2.3. Experimental Data

The experimental data we used are from the international authority of the database: PhysioNet/Fantasia [20]. In this database, there are 40 health subjects which have the same proportion of men and women, 20 of them are the elderly (65–85 years old, data name: f2o01m~f2o20m), the remaining subjects are the young (21–34 years old, data name: f2y01m~f2y20m). The ECG signal, continuous blood pressure signal, and respiration signal are recorded when the subjects under rest and watching the Fantasia movies to keep awake. However, only half of the subjects' blood pressure signals are recorded (data name: f2y01m-f2y10m, f2o01m-f2o10m). The data sampling frequency is 250 Hz, and the duration is 66 minutes.

The experimental data we used are the continuous blood pressure signals. Compared with the ECG signal, the blood pressure signal is uncalibrated. Therefore, the dynamic difference threshold method is used to calibrate the P waves [21], and the accuracy of the calibration is determined manually. Then, by making a first-order difference for the locations of the calibrated P waves, we will obtain a set of continuous PP intervals, which is PRV signal.

In reality, there are some singularities in PRV signal that have a bad effect on the signal processing results. A pretreatment method is employed to delete the singularities from these short-term PRV signals. The steps are as follows [22]:

*Step 1*. For the first datum PP(1) of PRV signal, if

(20) $$\begin{array}{c} \left|PP\left(1\right)-mean\left(PP\right)\right|>1.5\times std\left(PP\right), \end{array}$$

then PP(1) is a singularity and thus deleted. In (20), mean(PP), std(PP) are the mean and the standard deviation of a short-term PRV signal, respectively.

*Step 2*. For the *i*th datum PP(*i*) of PRV signal, if

(21) $$\begin{array}{c} \begin{array}{cc} PP\left(i\right)>1.3\times PP\left(i-1\right) & or\\ PP\left(i\right)<0.7\times PP\left(i-1\right), \end{array} \end{array}$$

then PP(*i*) is singular and deleted, where PP(*i* − 1) is the datum before PP(*i*).

## 3. Experimental Results

For simulating the process of PRV analysis in microcontroller system, the length of buffer is set to *N*_*w*_, which is the length of sliding window. In this study, the sliding window theory is used to data updating. When obtaining a new PRV datum, the data in buffer are analyzed with BSEA and SWIBSEA, and the performance of the two methods are compared.

**Figure 2:**
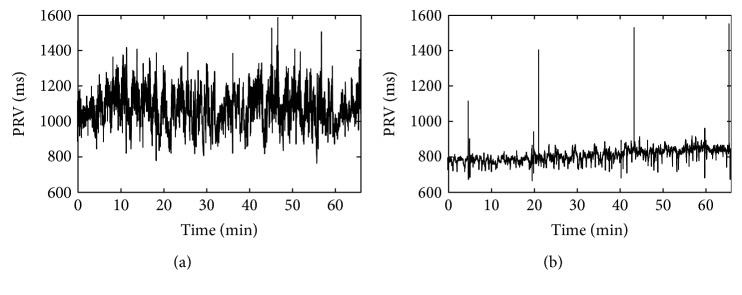
The PRV signal of a young subject (a) and an old subject (b).

The experimental data are continuous blood pressure signals that are used to generate PRV signals; the results of a young subject and an old subject are selected randomly and shown in Figure 2. With the individual differences in heart rate, the length of two PRV signals are different. For 66 minutes of data, the young one is 2793 points corresponding to the mean pulse rate is 56.1 beat per minute (bpm) and the old one is 4849 points corresponding to the mean pulse rate is 73.8 bpm.

In this study, the performance of SSEA and SWISSEA are quantitatively evaluated by mean square error (MSE) and program running time.

The MSE is defined as

(22) $$\begin{array}{c} MSE=\sqrt{\frac{1}{L}{\left[\overset{L}{\underset{i=1}{\sum }}BSE\left(i\right)-SWIBSE\left(i\right)\right]}^{2}}, \end{array}$$

where *L* is the length of entropy, BSE(*i*) is the BSE extracted by BSEA, and SWIBSE(*i*) is the BSE extracted by SWIBSEA.

For the program running time, we test the programs of SSEA and SWISSEA in MATLAB 2016a on a PC with i7-6700HQ CPU (2.60 GHz, 16 GB buffered RAM).

### 3.1. Comparison of BSEA and SWIBSEA

**Figure 3:**
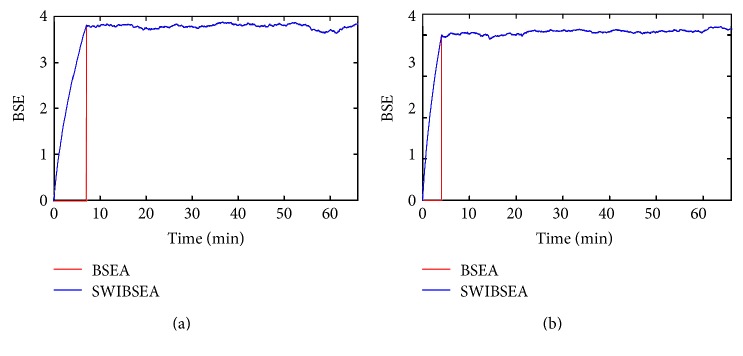
The comparison of BSEA and SWIBSEA for a young subject (a) and an old subject (b), when *α* = 0.5, *N*_*w*_ = 300, and *m* = 3.

The BSEA and the SWIBSEA are used to compute the entropy of the PRV signals in Figure 2. The results are shown in Figure 3.

When the total number of PRV data points in buffer is less than window width, *N* < *N*_*w*_, for BSEA, the BSE = 0 based on its theory; for SWIBSEA, this is the initial process, and we compute the entropy by iterative, and the value increases with *N*. When *N*_*w*_ ≥ *N*, the BSEA updates PRV data by sliding window and calculates BSE, while the SWIBSEA computes BSE by sliding window iteration; their entropies are shown in Figure 2. From the figure, it can be seen that their values are the same and their MSE = 0. The BSE of the young subject is 3.784 ± 0.050 (mean ± std), and the old subject is 4.056 ± 0.053, except for the entropy of the initial stage.

The time consumption of BSEA and SWIBSEA are 0.132 s and 4.769 s for the young subject and 0.192 s and 8.438 s for the old subject, respectively. For the two 66 minutes PRV signals, the time BSEA cost are 36 times and 44 times for SWIBSEA, respectively. Although the blood pressure signals of the old and the young subjects have the same length, but because of the individual differences, their pulse rates are different, and the PRV signal lengths are different. Thus, the time consumptions of the young and the old are difference. While compared with BSEA, SWIBSEA saves a lot of running time in computing BSE and keeps its values unchanged.

### 3.2. Comparison of BSEA and SWIBSEA under the Different Lengths of SSV

**Figure 4:**
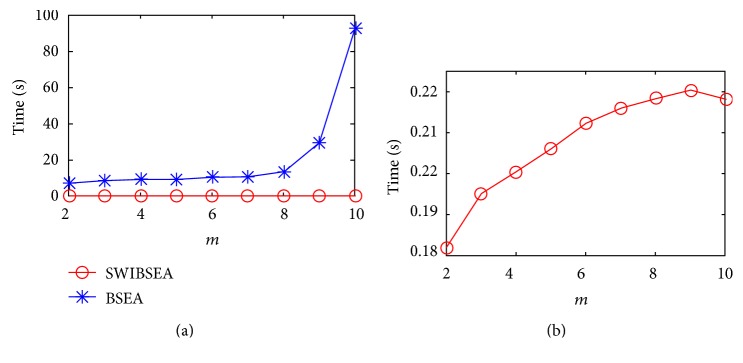
The time consumption of SWIBSEA (b) and BSEA (a) under the different length of SSVs for a young subject, when *N*_*w*_ = 300 and *α* = 0.5.

**Figure 5:**
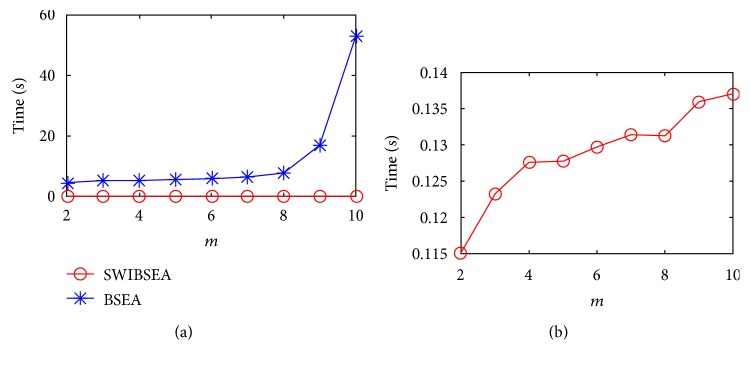
The time consumption of SWIBSEA (b) and BSEA (a) under the different length of SSV for an old subject, when *N*_*w*_ = 300 and *α* = 0.5.

According to the process of BSEA and SWIBSEA, the length *m* of the SSV has great influence on the running time of their program. The longer the length is, the more the heartbeat modes are represented by the SSVs. Therefore, keeping the width of sliding window and *α* unchanged (here, they are assigned to 300 and 0.5, respectively), we increase the value of *m* from 2 to 10, and the time consumption of these two methods for a young subject and an old subject are as shown in Figures 4 and 5.

Figure 4 shows the time consumption of the two method for the 66 minutes PRV signal of a young subject; Figure 4(a) is comparison of two method, and Figure 4(b) is the time consumption of SWIBSEA. When *m* increases from 2 to 10, the time they cost are all increased. The time consumption of SWIBSEA are from 0.182 s to 0.218 s, and that of BSEA are 42, 45, 45, 46, 50, 51, 62, 134, and 426 times for SWIBSEA. The growth rate of BSEA is 85.317 s, while that of SWIBSEA is only 0.036 s.

Figure 5 is the time consumption of an old subject. When *m* increases from 2 to 10, the time consumption of SWIBSEA are from 0.115 s to 0.137 s, and that of BSEA are 40, 42, 42, 44, 46, 49, 60, 126, and 388 times for SWIBSEA. The growth rate of BSEA is 48.524 s, while that of SWIBSE is only 0.022 s. Although the time consumption of the two methods are all increased, but the increase of SWIBSEA is far less than that of BSEA, SWIBSEA will save much more running time of a program.

**Figure 6:**
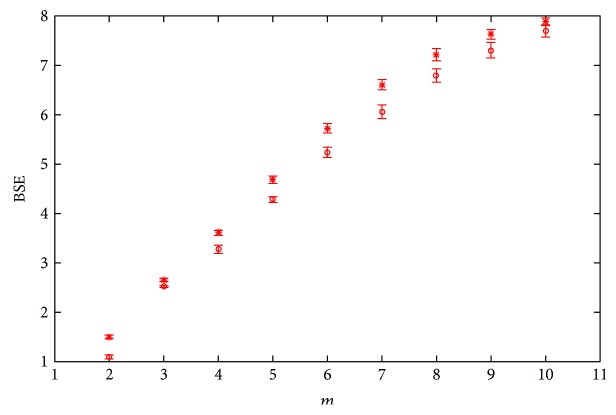
The BSE under different *m*'s, the results are shown with mean ± std, *N*_*w*_ = 300 and *α* = 0.5. “∗” is the BSE of old subjects; “o” is the BSE of young subjects.

The variation of *m* will cause some changes of the SSVs in the window, which inevitably causes the change of the BSE value. As is shown in Figure 6, when *m* increases from 2 to 10, the entropies of young subjects and old subjects are increased. There are significant differences between BSEA and SWIBSEA (*P* < 0.001, two-sample *t*-test), and the increase of *m* does not affect the difference between the young and the elderly.

### 3.3. Comparison of BSEA and SWIBSEA under Different *N*_*w*,*s*_

**Figure 7:**
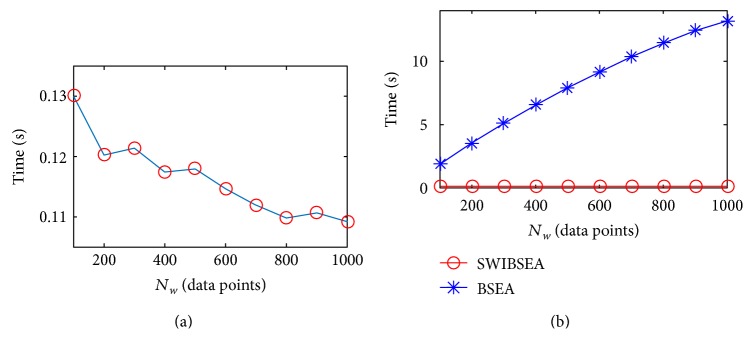
The time consumption of SWIBSEA (a) and BSEA (b) under the different *N*_*w*_ for a young subject, when *m* = 3, *α* = 0.5.

**Figure 8:**
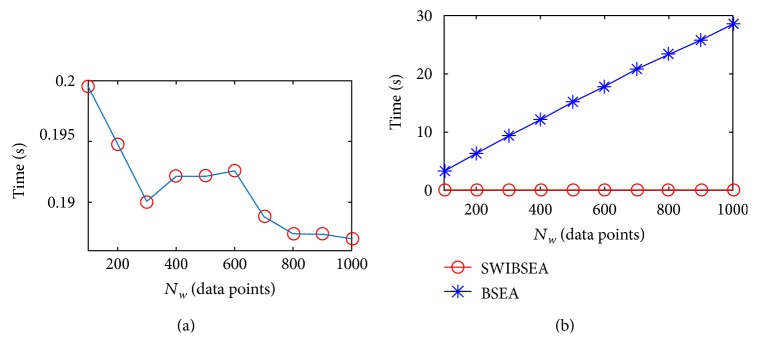
The time consumption of SWIBSEA (a) and BSEA (b) under the different *N*_*w*_ for an old subject, when *m* = 3, *α* = 0.5.

The width *N*_*w*_ of sliding window is corresponding to the length of buffer in a microcontroller system, and the range of *N*_*w*_ is varied from several minutes PRV data points (short-term PRV signal) to several hours data points (long-term PRV signal), or even to 24 hours PRV data points. The short-term PRV signal is used to derive some changes in the autonomic nervous system within a short time, and the long-term PRV signal is used to reflect the long time and slow changes of the autonomic nervous system. They have potential to apply in a clinic. However, with the increase of *N*_*w*_, the time consumption will increase inevitably. The results are shown in Figures 7 and 8. The values of *m* and *α* are chosen randomly; here, *m* = 3, *α* = 0.5. Then, the *N*_*w*_ increases from 100 to 1000 data points with the interval of 100. For the PRV signal of the young subject (in Figure 7), the time SWIBSEA used are from 0.1301 s to 0.1093 s, respectively. The time BSEA used are 15, 30, 42, 56, 67, 80, 93, 104, 112, and 121 times for SWIBSEA. For the PRV signal of the old subject (in Figure 8), the time SWIBSEA used are from 0.199 s to 0.185 s, respectively. The time BSEA used are 17, 33, 49, 63, 79, 93, 110, 124, 138, and 153 times for SWIBSEA. The increase of BSEA from *N*_*w*_ = 100 to *N*_*w*_ = 1000 are 25.188 s, but that of SWIBSEA decreases and the decrease is only 0.014 s. Because the SWIBSEA computes entropy with iterative and only updates the related variables of PP(1) and PP(*N*_*w*_), the BSEA needs to update all the variables in sliding window.

**Figure 9:**
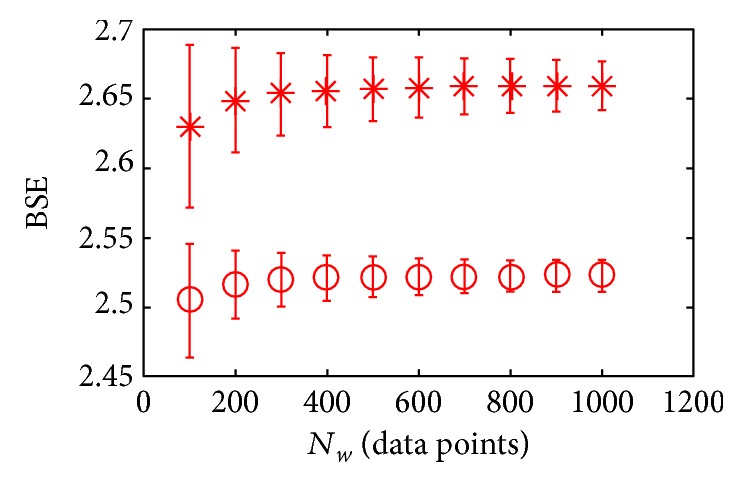
The BSE under different *N*_*w*_. The results are shown with mean ± std. *m* = 3 and *α* = 0.5. “∗” is the BSE of an old subject; “o” is the BSE of a young subject.

The change of *N*_*w*_ will have influence on BSE, as is shown in Figure 9. When *N*_*w*_ increases from 100 to 1000 data points, the difference of BSE between the young subject and the old subject are more and more larger.

## 4. Discussion

**Figure 10:**
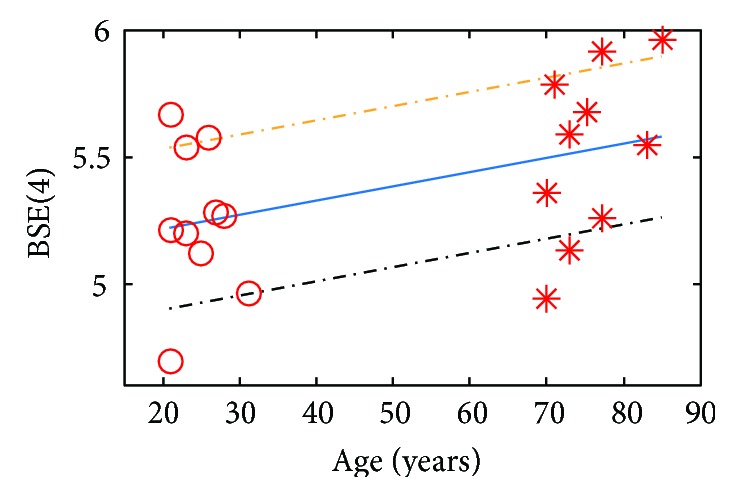
The SSE results of young subjects and old subjects. “o” is the young subjects, and “∗” is the old subjects. Solid line is the linear fitting result of BSE changed with age. Chain dotted line is the 95% confidential region.

BSEA, as a nonlinear method, has been employed to HRV signal analysis. Li and Ning [17] used BSEA to analyze the short-term HRV (500 data points) extracted from ECG signals of the PhysioNet/fantasia. When *m* = 4, *N*_*w*_ = 500, and*α* = 0.2, the results of BSEA show that the entropy increases with aging. The entropies are tested by two-sample *t*-test and *P* < 0.05. If the BSE of healthy young subjects represent the best physiological state of the human body, the BSE of healthy old subjects deviated from that of the old subjects, which indicates that normal aging can lead to some function degradation of the body's control system. We use SWIBSEA to analyze the HRV signals in [17] and get the same results as [17]. Similar to the HRV signal, the PRV signal is also generated from heartbeat. When *m* = 4, *N*_*w*_ = 500, and *α* = 0.2, the BSE of PRV signals are shown in Figure 10, and the entropies in the figure are the mean of BSE which are derived by SWIBSEA. The two-sample *t*-test of *P* = 0.008 and <0.05. We do the linear fitting of the entropies by aging, as shown on the solid line, the entropies increase with aging, the same as the result of short-term HRV signals analysis.

BSE has been effectively used for HRV signals analysis. In this study, on the basis of BSEA and with the theory of sliding window iterative, we proposed SWIBSEA for improving the computing efficiency of BSEA. Different from BSEA, it is not necessary to process all the data in buffer after date updating with sliding window for SWIBSEA. For example, when the buffer cache is 1024 bytes, *m* = 5. For BSEA, it needs more than 8169 bytes of memory space to store intermediate variables, that is, *N*_*w*_ = 1024 bytes for storing PRV data, *N*_*w*_ − *m* + 1 = 1024 − 5 + 1 = 1020 bytes for storing {BS(*i*)}, (*N*_*w*_ − *m* + 1) × *m* = (1024 − 5 + 1) × 5 = 5100 bytes for storing SSVs, 4*^m^* = 1024 bytes for storing the number of different SSVs, and 1 byte for storing BSE. Meanwhile, it also needs *N*_*w*_ − 1 = 1023 times shifting operations to update data; *N*_*w*_ − *m* + 1 = 1024 − 5 + 1 = 1020 times RMS operations to compute BS; 1020 times loops and (*N*_*w*_ − *m* + 1) × *m* × 4 = (1024 − 5 + 1) × 5 × 4 = 20400 times comparison operations to construct SSVs; *N*_*w*_ × *m* = 1024 × 5 = 5120 times comparison operations to updating {*n*(*j*)}; 1024 times multiplications for computing {*p*(*j*)}; 1024 times multiplications, 1024 times logarithms, and 1023 times additions for getting BSE. However, For SWIBSEA, it only needs 2061 bytes, that is, 1024 bytes for buffering PRV data, 2 bytes for storing BS_1_ and BS_*Nw*−*m*+2_, 2^∗^*m* = 10 bytes for storing {*S*_1_(*j*)} and {*S*_*N*_*w*_−*m*+2_(*j*)}, 4*^m^* = 1024 bytes for storing {*n*(*j*)}, and 1 byte for storing BSE. Meanwhile, it needs 1023 times shifting operations to update PRV signal, and 2 times RMS operations to compute BS_1_ and BS_*Nw*−*m*+2_, *m*^∗^4^∗^2 = 40 times comparison operations to compute {*S*_1_(*j*)} and {*S*_*N*_*w*_−*m*+2_(*j*)}, at most 9 times multiplications, 3 times logarithms, and 6 times additions to compute BSE (sometimes, it does not need to update the BSE). Since the storing addresses of {*n*(*j*)} are generated by encoding, no loops and comparison operations are required when update {*n*(*j*)}. Compared with BSEA, the SWIBSEA saves 6108 bytes buffer space. In addition, the sliding window iterative theory is used by the SWIBSEA, thus, its time consumption are reduced, and it can be engaged in PRV signal analysis in real time.

Moreover, the proposed method significantly reduces the time consumption and buffer cache, and can be used for both short-term and long-term PRV signals (in Section 3.3) by adjusting the width of the sliding window. The length of PRV signal only determines the initialization time; after the initialization, the BSE can be calculated by iterative, and the time consumption between the short-term PRV signal and the long-term PRV signal has not significantly increased.

## 5. Conclusion

In this study, the sliding window iterative theory is used to improve the BSEA, and the SWIBSEA is proposed and employed to analyze the data of healthy young and old subjects from MIT/PhysioNet/Fantasia database. The results show that compared with BSEA, the SWIBSEA reduces the computing time and saves the buffer cache while keeping the BSE unchanged. Meanwhile, by adjusting the width of sliding window, the SWIBSEA can analyze the long-term and short-term PRV signals in real time. The experimental results show that the BSE increases with aging, and normal aging leads to some functions degradation of the control system. Therefore, the SWIBSEA could be employed in some wearable and portable devices for analyzing dynamic PRV signal in real time.
